# The Peptide LLTRAGL Derived from *Rapana venosa* Exerts Protective Effect against Inflammatory Bowel Disease in Zebrafish Model by Regulating Multi-Pathways

**DOI:** 10.3390/md22030100

**Published:** 2024-02-22

**Authors:** Yongna Cao, Fenghua Xu, Qing Xia, Kechun Liu, Houwen Lin, Shanshan Zhang, Yun Zhang

**Affiliations:** 1 Biology Institute, Qilu University of Technology (Shandong Academy of Sciences), Jinan 250103, China; caoyn123987@163.com (Y.C.); xfh20171215@163.com (F.X.); xiaq@sdas.org (Q.X.); hliukch@sdas.org (K.L.); franklin67@126.com (H.L.); 2 Engineering Research Center of Zebrafish Models for Human Diseases and Drug Screening of Shandong Province, Jinan 250103, China; 3 Research Center for Marine Drugs, State Key Laboratory of Oncogenes and Related Genes, Department of Pharmacy, School of Medicine, Shanghai Jiao Tong University, Shanghai 200127, China

**Keywords:** *Rapana venosa*, LLTRAGL, inflammatory bowel disease, 2,4,6 trinitrobenzene sulfonic acid, zebrafish, transcriptome analysis, NOD-like receptor, necroptosis

## Abstract

Inflammatory bowel disease (IBD) is a chronic inflammatory bowel disease with unknown pathogenesis which has been gradually considered a public health challenge worldwide. Peptides derived from *Rapana venosa* have been shown to have an anti-inflammatory effect. In this study, peptide LLTRAGL derived from *Rapana venosa* was prepared by a solid phase synthesis technique. The protective effects of LLTRAGL were studied in a 2,4,6-trinitrobenzene sulfonic acid (TNBS)-induced zebrafish colitis model. The underlying mechanisms of LLTRAGL were predicted and validated by transcriptome, real-time quantitative PCR assays and molecular docking. The results showed that LLTRAGL reduced the number of macrophages migrating to the intestine, enhanced the frequency and rate of intestinal peristalsis and improved intestinal inflammatory damage. Furthermore, transcriptome analysis indicated the key pathways (NOD-like receptor signal pathway and necroptosis pathway) that link the underlying protective effects of LLTRAGL’s molecular mechanisms. In addition, the related genes in these pathways exhibited different expressions after TNBS treatment. Finally, molecular docking techniques further verified the RNA-sequencing results. In summary, LLTRAGL exerted protective effects in the model of TNBS-induced colitis zebrafish. Our findings provide valuable information for the future application of LLTRAGL in IBD.

## 1. Introduction

Inflammatory bowel disease (IBD) is a chronic inflammation in the gastrointestinal tract which has been categorized into ulcerative colitis (UC) and Crohn’s disease (CD) [1,2]. According to statistics, about 6.8 million cases of IBD were documented worldwide [3]. Due to unpredictable and highly variable symptoms, IBD presents a major healthcare burden of global morbidity, with the highest prevalence in Europe and North America, and rising incidence in Asia [4]. The treatment protocols for IBD mainly rely on medication therapy or surgery [4,5]. In addition to the preferred choice of anti-inflammatory drugs (antibiotics), biological agents (primarily anti-TNF-α) and immunosuppressants are also used for the treatment of IBD [4,6]. However, long-term use of these medications is often not safe for the body due to their potential side effects. In this regard, the search for new sources of drugs to improve the clinical safety and efficacy of IBD treatment remains essential.

Natural products derived from marine organisms often exhibit novel chemical structures and unique physiological effects because the living environment is different from terrestrial organisms. Marine peptides which can be isolated from algae, molluscs, fish and marine by-products [7,8,9] show multi-bioactivities, such as anti-inflammatory, antihypertensive, antioxidant, antimicrobial and neuroprotective activities, etc. [8,10,11,12,13,14]. Therefore, peptides have become important resources for new drugs, health foods, special biological functional materials and cosmetics in recent years [15]. *Rapana venosa* (*Rv*) is an important marine snail which exhibits an increasing nutritional and economic importance [16]. Some studies have demonstrated that hemocyanin and peptides from *Rv* exhibited antimicrobial and antitumor activities [14,17,18]. Additionally, a previous study in our lab indicated that peptides from *Rv* could significantly improve inflammation damage in zebrafish induced by metronidazole. Among six peptides which are disclosed in the China patent (Patent NO: 202010313278X), LLTRAGL showed positive anti-inflammatory activities without obvious side effects on zebrafish. Furthermore, in a recent study, it was proved that the anti-inflammatory peptides derived from food-resource phycocyanin had better anti-IBD activity [19]. This study provides a possibility for the study of the same food-derived anti-inflammatory peptides in anti-IBD with safety and effectiveness. However, the anti-IBD activity and fundamental mechanism of action of LLTRAGL is still unclear.

Zebrafish is an ideal model system for human disease and drug development with the advantages of high reproducibility, rapid development, small size, high throughput, low cost and high genetic and morphological similarity with the human counterpart [20,21]. Many studies have reported that zebrafish (Danio rerio) larvae have emerged as a useful tool for the screening and exploration of potential drugs in the treatment of IBD and gastrointestinal diseases [22,23].

Studies have proved that inflammatory reactions are a crucial factor in the development of IBD. Hence, peptides from *Rv* may be promising therapeutics for IBD. Nonetheless, there is little information about peptides from *Rv* on the application in IBD. In this study, the peptide LLTRAGL, which was purified and characterized from the enzymatic *Rv* tissue, was evaluated for its potential to alleviate IBD symptoms in 2,4,6 trinitrobenzene sulfonic acid (TNBS)-induced zebrafish for the first time; the potential molecular mechanisms of LLTRAGL were investigated.

## 2. Results

### 2.1. Effect of Peptide LLTRAGL on the Migration of Macrophages in Zebrafish Juvenile Treated with TNBS

**Figure 1:**
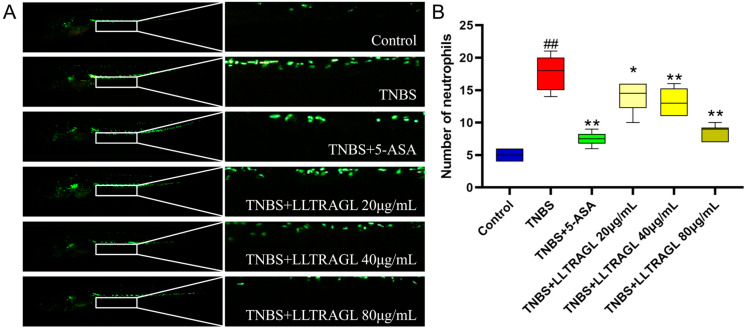
Effect of peptide LLTRAGL on TNBS-induced migration of zebrafish to intestinal macrophages. (**A**) Typical fluorescence images of Tg (*zlyz*: *EGFP*) macrophage migration in transgenic zebrafish juveniles. (**B**) Box chart showing the number of macrophages migrating from zebrafish larvae to the intestine. (**A**) the image on the right is an enlarged image of the white box on the left. Compared with the blank group, ## *p* ≤ 0.01; compared with the TNBS group, * *p* ≤ 0.05 and ** *p* ≤ 0.01.

To assess the effects of the peptide LLTRAGL on the development of colitis, a colitis model in zebrafish was established by inducing TNBS. The safe concentrations of the peptide were screened from 10 to 640 μg/mL by using a zebrafish model (Supplementary Figure S1). LLTRAGL could inhibit the migration of macrophages to the intestine at 20 μg/mL, while the effect of inhibiting the migration of macrophages to the intestine at 80–320 μg/mL was similar. Furthermore, after treating zebrafish with LLTRAGL at 640 μg/mL, they were all dead. Therefore, the dosages of LLTRAGL were chosen at 20, 40 and 80 μg/mL. Later, the colitis zebrafish larvae were treated with LLTRAGL (20, 40, 80 μg/mL) and 5-ASA (1 μg/mL) for 24 h, respectively. According to the results in Figure 1, TNBS significantly induced colitis in zebrafish with increasing numbers of neutrophils cells in the intestines and enlarging the damaged intestinal area, which was compared to the control group. However, compared with the TNBS group, the positive drug and LLTRAGL significantly improved the intestinal inflammation of zebrafish (*p* ≤ 0.01 and *p* ≤ 0.05). LLTRAGL can reduce the number of macrophages in the intestine with dose dependency. In particular, at the high dose group (80 μg/mL) the peptide could significantly reduce the number of macrophages migrating to the intestine (*p* ≤ 0.01). The data suggest that LLTRAG is effective in ameliorating colitis and reducing inflammatory responses.

### 2.2. Effect of Peptide LLTRAGL Promote Gastrointestinal Motility in Zebrafish

**Figure 2:**
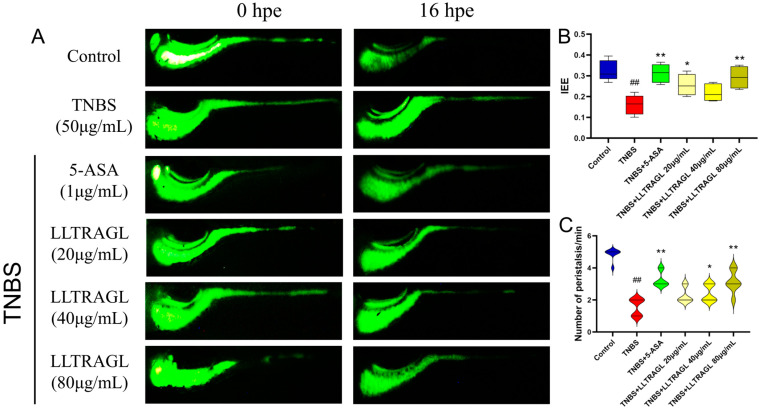
LLTRAGL improves TNBS-induced intestinal peristalsis damage. (**A**) Typical fluorescence images of intestinal efflux rate (IEE) of wild zebrafish juveniles. The blue box shows that after staining with calcein for 1.5 h, the drug was administered for 16 h. (**B**) Box chart showing the intestinal efflux rate of zebrafish juveniles. (**C**) Violin chart showing the number of intestinal peristalses in zebrafish juveniles. Compared with the blank group, ## *p* ≤ 0.01; compared with the TNBS group, * *p* ≤ 0.05 and ** *p* ≤ 0.01.

It is well known that colitis causes gastrointestinal dysfunction and influences the intestinal peristalsis capacity. To further investigate the potential anti-inflammatory effect of peptide in IBD-induced zebrafish colitis, the promoted gastrointestinal motility was evaluated by using a fluorescent dye solution. TNBS-treated zebrafish showed strong fluorescence density in the intestine (Figure 2A). This result indicated that TNBS significantly depressed the intestinal efflux efficiency (IEE) and the intestinal peristalsis frequency of the control group (*p* ≤ 0.01, Figure 2B,C). However, the inhibitory effect could be significantly ameliorated by 5-ASA (*p* ≤ 0.01, Figure 2). Furthermore, LLTRAGL treatment could also alleviate the depressed IEE and promote the gastrointestinal peristalsis frequency of zebrafish compared with the TNBS-treated zebrafish, especially at the concentration of 80 μg/mL, demonstrating the therapeutical effects of the peptide on TNBS-induced IBD (*p* ≤ 0.01, Figure 2).

### 2.3. Effect of Peptide LLTRAGL on TNBS-Induced Pathological Changes and Ultrastructure of Intestinal

**Figure 3:**
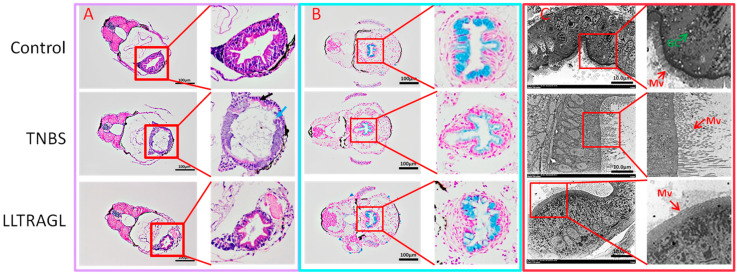
Effects of the peptide on TNBS-induced intestinal tissue pathology and ultrastructure of zebrafish. (**A**) H&E staining of intestinal tissues. Black arrow: sparse arrangement of cells; Blue arrow: Mucosal layer necrosis, cell lysis, enhanced cytoplasmic basophilia, disappearance of intestinal folds. Scale bar is 100 µm. (**B**) Alcian blue (AB) staining of intestinal tissues. Scale bar is 100 µm. (**C**) Electron microscopy of intestinal ultrastructure. Red arrow: intestinal microvilli status; Mv represents microvilli; Green arrow: goblet cell state; GC stands for goblet cell. Scale bar is 10.0 µm.

The zebrafish intestinal histopathological changes are shown in Figure 3. The hematoxylin and eosin (H&E) images in Figure 3A clearly showed that the intestinal epithelial cells of larvae in the control group showed clear structures of mucosa, muscularis propria, and serosa, and columnar epithelial cells were neatly arranged. TNBS treatment caused a decrease in intestine folding, goblet cells, columnar epithelial cells, and an increase in inflammatory cell infiltration, cilia defects and mucus volumes. In contrast, the LLTRAGL treatment group can significantly improve the damage caused by TNBS, and the number of goblet cells and columnar epithelial cells were increased. Furthermore, the gut tissue morphology was closely similar to that of normal zebrafish. The Alcian blue (AB) staining was performed to determine the mucus levels in the intestinal goblet cells. According to the results shown in Figure 3B, compared with the control group, the mucous in the intestines of zebrafish was significantly reduced after TNBS treatment. However, an obvious recovery of mucin was observed after peptide treatment. Subsequently, the ultrastructure of the intestinal tissue of zebrafish was shown in Figure 3C. The intestinal microvilli of zebrafish in the control group were arranged neatly and were uniform in thickness. However, disrupted and shortened microvilli, defective, tight and adherent junctions of intestinal epithelial cells, and decreased goblet cell numbers were observed in TNBS-treated zebrafish. LLTRAGL treatment could recover the injured intestinal microvilli and reverse them to a state similar to that of normal zebrafish.

### 2.4. Transcriptome Analysis of Peptide LLTRAGL in Improving TNBS-Induced Inflammatory Bowel Disease Damage

#### 2.4.1. Differentially Expressed Genes (DEGs) by Transcriptome Analysis

**Figure 4:**
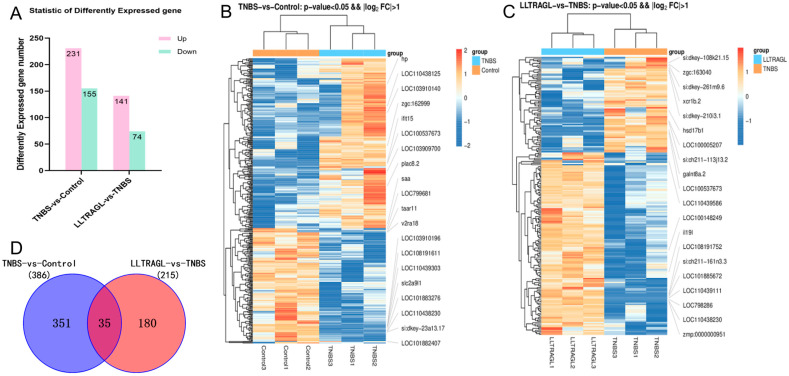
The differentially expressed genes among the control group, TNBS group and peptide treatment group in RNA-Seq. (**A**) Statistical histogram of differentially expressed genes in TNBS vs. Control and LLTRAGL vs. TNBS. (**B**) TNBS vs. Control differential gene grouping cluster diagram. (**C**) LLTRAGL vs. TNBS differential gene grouping cluster diagram. (**D**) Venn diagram of common and unique differentially expressed genes between the TNBS vs. Control and LLTRAGL vs. TNBS comparative groups. The red color in the figure represents genes encoding relatively high expression proteins, while the blue color represents genes encoding relatively low expression proteins.

In order to further analyze the effects of LLTRAGL on the transcription level, the mRNAs from the control group, TNBS treatment group and peptide treatment group were sequenced. A total of 386 DEGs (the absolute value of log2FoldChange ≥ 1; *p*-value < 0.05) were identified between the TNBS group and control group. Among these genes, 231 were up-regulated and 155 were down-regulated genes. In the peptide-treated group, there were 215 DEGs compared to the TNBS group, with 141 DEGs up-regulated and 74 DEGs down-regulated (Figure 4A). The top 10 up- and down-regulated DEGs for each comparison are displayed in Figure 4B,C (Supplementary Table S1). Venn analysis showed that the two comparison groups had 35 DEGs in common (Figure 4D, Supplementary Table S2).

#### 2.4.2. GO Term Enrichment Analysis

**Figure 5:**
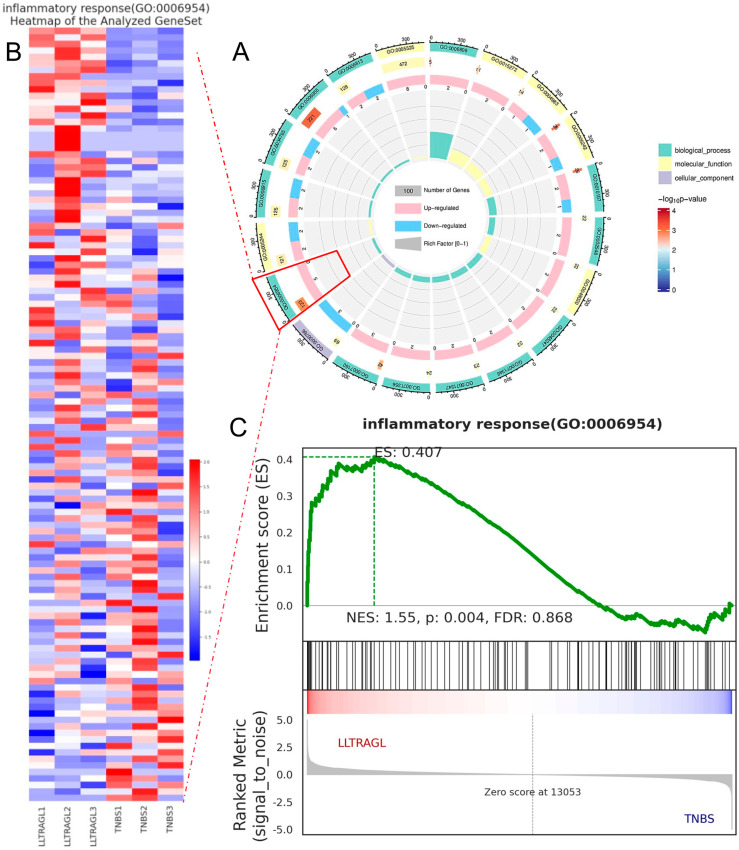
GO enrichment analysis of DEGs and GSEA of all tested genes. (**A**) Circle plots of the distribution of DEGs in different GO categories. The circle chart shows the distribution of DEGs in the molecular function (MF), biological process (BP), and cellular composition (CC) categories. (**B**) Grouping clustering diagram of inflammatory response in the GSEA analysis. (**C**) Analysis Results of the Inflammatory Response Gene Set, mainly including the distribution map of enrichment score (ES), gene distribution map of gene set, and distribution map of the measurement and control bar sorting matrix.

Based on the above DEGs, the GO function of differentially expressed genes between TNBS-treated zebrafish and LLTRAGL treatment zebrafish was analyzed by Gene Set Enrichment Analysis (GSEA) to study the function of DEGs. In the GO functional concentration analysis (Figure 5A), the biological process of zebrafish enrichment in the LLTRAGL-treated group was mainly related to the inflammatory response compare with the TNBS group (Supplementary Table S3). According to GSEA and cluster mapping, the expression of inflammatory response-related genes in LLTRAGL-treated zebrafish was significantly reversed, compared with that of the TNBS-treated group (Figure 5B,C, Supplementary Table S4).

#### 2.4.3. KEGG Pathway Enrichment Analysis

**Figure 6:**
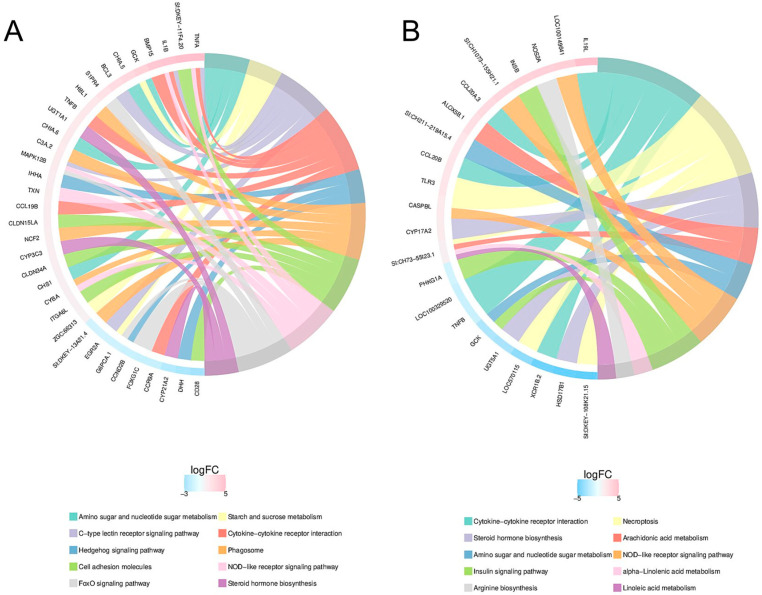
KEGG pathway enrichment analysis of DEGs. (**A**) KEGG-enriched top 10 chord diagram of TNBS vs. Control. (**B**) KEGG-enriched top 10 chord diagram of LLTRAGL-vs-TNBS. KEGG, Kyoto Encyclopedia of Genes and Genomes.

**Figure 7:**
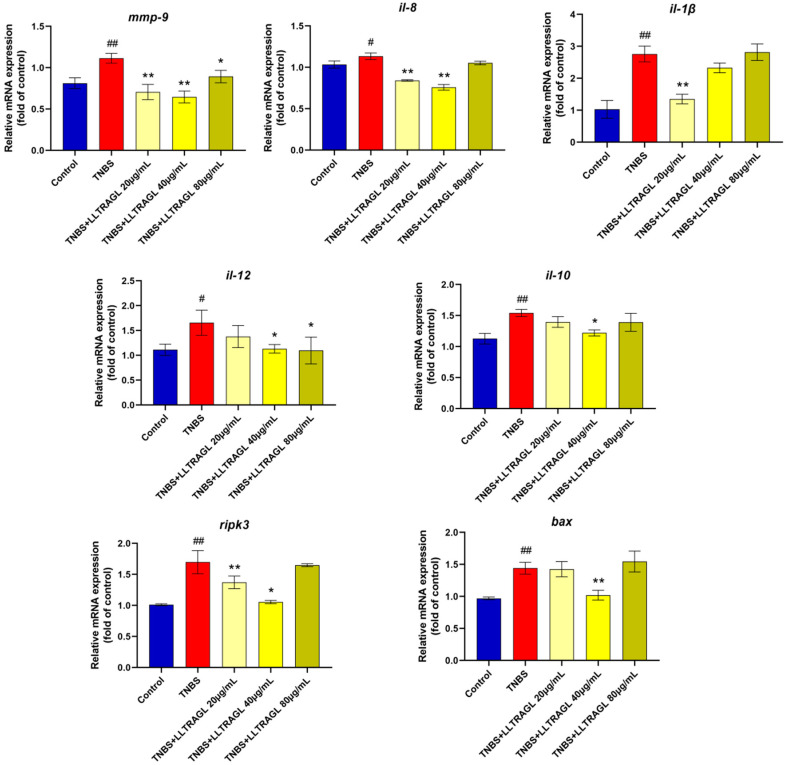
Effect of LLTRAG on the inflammation-related gene expression in zebrafish, determined by RT-PCR. Compared with the blank group, # *p* ≤ 0.05 and ## *p* ≤ 0.01; compared with the TNBS group, * *p* ≤ 0.05 and ** *p* ≤ 0.01.

KEGG pathway analysis showed that the up-regulated genes between the TNBS and control group involve the top 10 pathways, including the NOD-like receptor signaling pathway, necroptosis, C-type lectin receptor signaling pathway and cytokine–cytokine receptor interaction. The down-regulated genes involve the top 10 pathways, including Neuroactive ligand–receptor interaction, Phenylalanine, tyrosine and tryptophan biosynthesis, Hedgehog signaling pathway and FoxO signaling pathway (Figure 6A, Supplementary Table S5). For the KEGG pathway analysis between the LLTRAGL and TNBS group, the up-regulated genes involved in the top 10 pathways are the NOD-like receptor signaling pathway, necroptosis, cytokine–cytokine receptor interaction and the MAPK signaling pathway, etc. The down-regulated genes involve the key pathways, including necroptosis and cytokine–cytokine receptor interaction signaling pathway (Figure 6B, Supplementary Table S5). KEGG pathway analysis showed that the differentially expressed genes involved inflammation-related pathways, of which NOD-like receptor signaling pathway and necroptosis pathway were the most significant pathways. Subsequently, we further validated the accuracy of the RNA-sequencing analysis by a quantitative RT-PCR of seven gene expression levels, which were related to the inflammation. Results showed that the transcription levels of *mmp9*, *il8*, *il1β*, *il12*, *il10*, *ripk3* and *bax* genes were consistent with the results of the transcriptome sequencing (Figure 7). These results indicated that the LLTRAGL peptide might alleviate zebrafish IBD via regulating multiple inflammation-related pathways.

### 2.5. LLTRAGL Alleviates Inflammatory Bowel Disease Damage through the NOD-Like Receptor Signaling Pathway and Necroptosis Signaling Pathway

**Figure 8:**
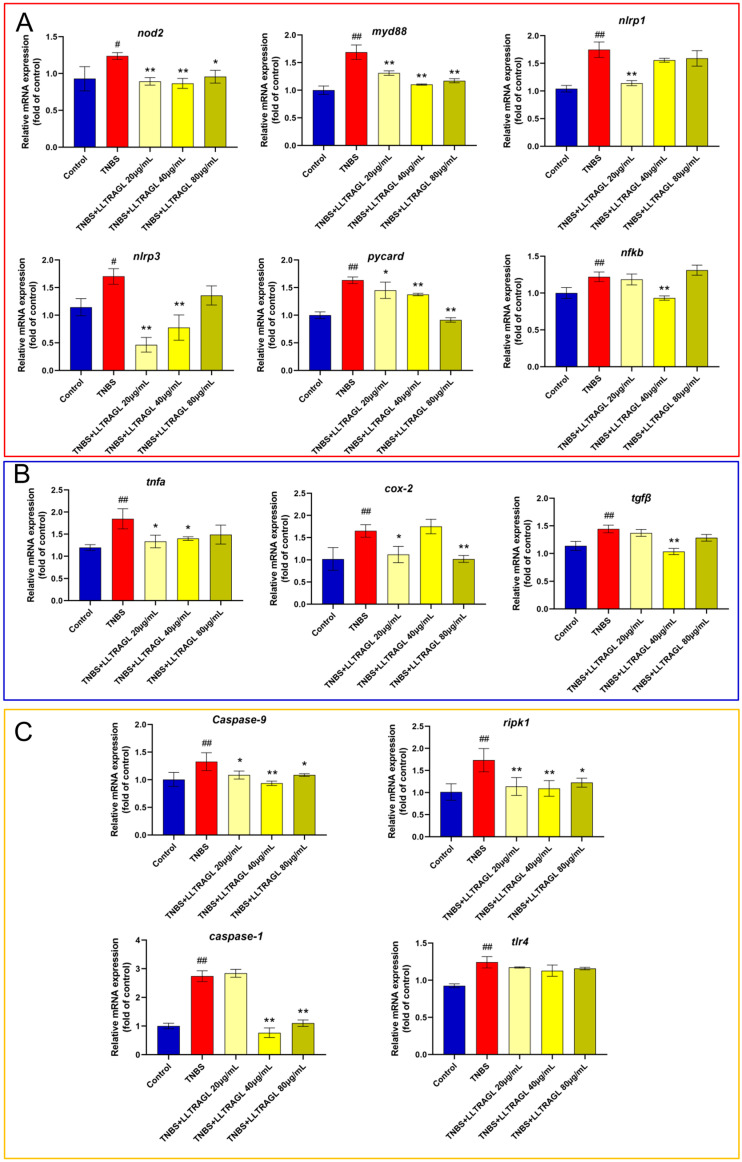
Effect of LLTRAG on gene expression in TNBS-induced colitis in zebrafish. (**A**) NOD-like receptor signal pathway-related genes. (**B**) Inflammatory factors-related genes. (**C**) necroptosis signal pathway-related gene. Compared with the blank group, # *p* ≤ 0.05 and ## *p* ≤ 0.01; compared with the TNBS group, * *p* ≤ 0.05 and ** *p* ≤ 0.01.

NOD-like receptor and necroptosis signaling pathways exist in the KEGG concentration of mRNA-seq in colitis zebrafish treated with LLTRAGL. We think these two signaling pathways may be necessary for LLTRAGL to improve inflammatory bowel disease. To test our hypothesis, we quantitatively detected the expression of the NOD-like receptor and necroptosis signaling pathway-related proteins in the untreated zebrafish blank group, peptide treatment group and TNBS group. Compared with the control blank group, the TNBS group showed a significant increase in the expression of *nod2*, *myd88*, *nlrp1*, *nlrp3*, *pycard*, *nfkb*, *tnfa*, *cox-2*, *tgfβ* and *il-10* genes; moreover, *caspase-9*, *ripk1*, *caspase-1* and *tlr4* genes, which are related to necroptosis, are also up-regulated. After LLTRAGL treatment, the up-regulated gene expression by TNBS could significantly reverse (Figure 8). Combined with the mRNA-seq analysis results, we found that LLTRAGL down-regulated the expression of genes in the NOD-like receptor signal pathway and necroptosis signal pathway, thus playing a role in treating IBD in zebrafish.

### 2.6. Insight into Molecular Docking Simulation

To predict the binding affinity of LLTRAGL to the target, molecular docking was performed by selecting target genes related to the NOD-like receptor signaling pathway and necroptosis signaling pathway as receptors while using 5-ASA as a positive control to assist in validating the pharmacological effects. Molecular docking was performed using LLTRAGL and 5-ASA as ligands, and the key targets of the NOD-like receptor signaling pathway and necroptosis signaling pathway genes PYCARD (6KI0), IL-8 (6WZL), IL-1β (7Z3W), Caspase-9 (5WVC) and RIPK1 (7YDX) as receptors and the docking results are shown in Table 1.

Compared with the positive control 5-ASA, the binding energy of LLTRAGL to PYCARD, IL-8, IL-1β, Caspase-9 and RIPK1 proteins all had lower CDOCKER INTERACTION ENERGY, with the highest binding energy of −89.7209 and the lowest binding energy of 138.7620 KJ/mol, indicating that LLTRAGL had a good affinity with all target genes. The LLTRAGL peptide combined with receptors through the interaction forces of hydrogen bonds, as well as the electrostatic and hydrophobic forces. LLTRAGL formed more stable hydrogen bonds and hydrophobic forces with the surrounding residues in the active site of the target proteins than 5-ASA. (Figures S2 and S3)

## 3. Discussion

IBD is a chronic and recurrent inflammatory disease of the gastrointestinal tract [20]. As a typical inflammatory disease, research indicates that immune cell types are involved in the pathogenesis of IBD [24]. However, the mechanism of IBD pathogenesis is extremely complex and unclear, which has limited the development of effective treatments. Therefore, new therapeutic strategies for IBD are urgently needed. A growing interest has been focused on compounds obtained from natural resources.

The effects of peptide LLTRAGL derived from *Rapana venosa* on TNBS-induced colitis in zebrafish were investigated. Studies had indicated that LLTRAGL could reduce the inflammatory responses in the TNBS-induced zebrafish model of colitis by suppressing the number of neutrophil cells in the intestine, alleviating the damage in the intestinal area and promoting gastrointestinal motility. Furthermore, the ameliorative effect of the peptide on TNBS-induced colitis may be associated with the modulation of a NOD-like receptor signal pathway and necroptosis pathway-associated genes. Molecular docking further proved the efficacy of the interaction between LLTRAGL and key proteins in the inflammation-related signaling pathways.

TNBS administration can induce the IBD model with the hallmark aspects of human IBD, including the induction of pro-inflammatory pathways, the occurrence of neutrophils and macrophages in the intestine, and reduce the intestinal peristalsis [25,26]. An increase in the intestinal neutrophil numbers and gastrointestinal motility disorders in zebrafish were found in TNBS-treated zebrafish, which validated the applicability of TNBS for generating the colitis models [26]. After LLTRAGL treatment, the colitis symptoms of TNBS-induced zebrafish were significantly ameliorated.

The complete intestinal epithelium [27] and intestinal barrier [28] are crucial for maintaining normal intestinal physiological function. In this study, LLTRAGL and TNBS co-treatment zebrafish can improve the infiltration of inflammatory cells, decreasing goblet cells, and crypt damage. Furthermore, the similar intestinal morphology improvement effects of drugs with anti-IBD activities can be observed in the mouse model [28,29,30]. These suggested that the peptide LLTRAGL may reduce inflammatory bowel disease by improving the integrity of the intestinal epithelial tissue and intestinal barrier.

The transcriptome analysis is used to study gene expression at the RNA level. The annotation results of GO showed that the differential genes between LLTRAGL- and TNBS-induced colitis zebrafish were mainly enriched in biological processes, such as inflammatory response and cell apoptosis. As shown in the KEGG pathway analysis results, there were significant changes in NOD-like receptor signaling and necroptosis pathway in LLTRAGL treatment zebrafish and TNBS treatment zebrafish.

Numerous experiments in vitro and in vivo have indicated the production of anti-IBD functions by regulating NOD-like receptor signaling [31,32]. NOD1 and NOD2, as sensors of peptidoglycan and cellular stress and activators of multiple signaling pathways involved in immune response, are critical players in the resistance to inflammatory diseases [33]. NOD2 is involved in the recognition of infectious bacteria, induction of immune responses, and protection of the intestinal mucosal barrier from bacterial erosion through the activation of nuclear factor kappa B (NF-κB) [34]. NLRP1 is the first pattern recognition receptor (PRR) discovered to form an inflammasome, and genome-wide association studies have identified NLRP1 mutations linked with IBD [35]. The NLRP3 can produce IL-1β and IL-18 and initiate inflammatory processes [31]. In addition, IL-1β promotes the production of IL-22 and IL-10 to maintain epithelial integrity [36,37]. In the previous study, the genes related to the NOD-like receptor signaling pathway, include *myd88*, *nod2*, *nlrp1*, *nlrp3* and *pycard* were significantly up-regulated in TNBS treatment zebrafish. However, LLTRAGL could effectively reverse the up-regulation of the key genes in zebrafish.

Necroptosis is a cell death pathway that is supposed to be of importance in the pathogenesis of many diseases such as cancer, IBD and other intestinal diseases [38,39]. The transcriptome analysis of the colon tissue of DSS-induced colitis mice indicates that the necroptosis pathway plays an anti-inflammatory role in IBD [40]. In addition, the necrotic apoptotic pathway was also enriched in KEGG analysis according to the transcriptome analysis of TNBS zebrafish. The key genes involved in the necroptosis signaling pathway, *caspase-9*, *ripk1*, *ripk3*, *caspase-1*, *tlr4* and *bax*, were significantly up-regulated in TNBS treatment zebrafish. Significant reversals of gene expression levels were found in the peptide-treated zebrafish.

A combination of the transcription analysis and RT-PCR results demonstrates that the level gene expression of *pycard*, *il-8*, *il-1β*, *caspase-9* and *ripk1* is critical to the efficacy of IBD inhibition. Thus, the proteins PYCARD, IL-8, IL-1β, Caspase-9 and RIPK1 involved in the NOD-like receptor and necroptosis signaling pathway were selected as receptors [41,42,43]. Molecular docking was used to further verify the mRNA-sequencing results. The docking results showed that LLTRAGL had a lower binding energy compared to 5-ASA, indicating that LLTRAGL had a higher affinity for all five proteins, which further confirmed that the mechanism of LLTRAGL treatment for zebrafish colitis might be related to the activation of the NOD-like receptor signaling pathway and necroptosis signaling pathway.

In a comprehensive analysis of the phenotypic and mechanistic results, there were some differences in the trend of LLTRAGL. According to the phenotypic results in the zebrafish model, the group with the optimal concentration of 80 μg/mL was selected for transcriptome analysis. This phenomenon may be because LLTRAGL regulates the influence of changes in gene level first, then affects protein level, regulates various reactions in the body, and finally shows the reduction in macrophages. The 80 μg/mL concentration may be due to the body’s own regulation of gene overexpression.

## 4. Materials and Methods

### 4.1. Chemicals and Reagents

2,4,6-trinitrobenzenesulfonic acid (TNBS), 5-aminosalicylic acid (5-ASA), and calcein were purchased from Sigma-Aldrich (Shanghai, China). All other chemicals and reagents were of analytical grade from local commercial sources.

### 4.2. LLTRAGL Peptide Preparation

LLTRAGL peptide was isolated and purified from *Rv* enzymolysis protein by an activity-directed separation technique, then the amino acid sequence was identified by LC-MS omics. The LLTRAGL peptide consisted of Leu-Leu-Thr-Arg-Ala-Gly-Leu and had a molecular weight 742 Da. Its anti-inflammation activity was proved by the zebrafish model. According to the patent records and previous research studies, it was found that the observed inflammatory activity of the peptide is attributed to its low molecular weight, high proportion of hydrophobic and positively charged amino acids [44,45]. The synthetic peptide of LLTRAGL was used in the study (CelLmano Biotech Co., Ltd., Hefei, China) with a purity of 95%.

### 4.3. Zebrafish Maintenance

The wild-type AB strain and transgenic zebrafish (*zlyz*: *EGFP*) strain were used for animal experiments which were obtained from the Engineering Research Center of Zebrafish Models for Human Diseases and Drug Screening of Shandong Province (Jinan, China). The zebrafish embryos were kept under a constant temperature (28 ± 0.5 °C) and 14 h-light/10 h-dark cycles. The healthy zebrafish were paired with a sex ratio at 2:2 (female:male) overnight and embryos were collected the next morning. Then, zebrafish embryos were transferred into clean culture water (5 mM NaCl, 0.17 mM KCl, 0.33 mM CaCl_2_, and 0.33 mM MgSO_4_) containing 2 mg/L methylene blue solution after three washes [46].

### 4.4. TNBS-Induced Colitis Experiment

Colitis in zebrafish was induced by TNBS to evaluate the effects of LLTRAGL on mitigating the colitis according to the published literature [26]. The 3 dpf (days post-fertilization) zebrafish larvae of the Tg: *zlyz-EGFP* strain were randomly placed into 24-well plates (10 per well), and then randomly divided into six groups. Four parallel holes per group were set in the experiment. Except for the blank group, which was incubated in fish water without TNBS, the zebrafish in the other groups were incubated in water with TNBS (60 μM) under the same conditions. After 48 h of incubation, the positive control group and peptide treatment groups were added into 5-ASA and three different concentrations of peptide (20, 40, 80 μg/mL). Then, the zebrafish larvae were anesthetized with tricaine (0.2%, *w*/*v*) after 24 h of incubation. The number of neutrophil cells in the zebrafish intestines was used as an evaluation index which was observed and photographed via a microscope (Olympus, SZX2-ILLTQ, Tokyo, Japan).

### 4.5. Intestinal Efflux Efficiency Experiment

AB zebrafish embryos at 3 dpf were randomly divided into six groups; namely, the blank control group; the 5-ASA positive control group; the TNBS model group; and the low-, medium-, and high-dosage peptide groups, with 4 repeat holes per group (10 per well). Next, the zebrafish colitis model was constructed by exposing larvae to TNBS. After 48 h, each group was stained with 0.2% calcein solution for 1.5 h. Then the dye was removed by washing the zebrafish three times with phosphate-buffered saline (PBS). The intestinal fluorescence of zebrafish was determined by using an Olympus SZX16 fluorescence microscope (Tokyo, Japan) equipped with a digital color camera (DP2-BSW, cellSens Standard software 2.2). The integrated option density (IOD) of each zebrafish was analyzed using Image Pro-Plus 5.1. The average IOD of the control group was defined as IOD0, and the average IOD of the TNBS group was defined as IOD1. Then, the positive drug and different concentrations of peptide LLTRAGL (20, 40, 80 μg/mL) were added into positive control group and peptide treatment groups. All the zebrafish were cultured in a dark environment for 16 h. After that, the fluorescence intensities of the zebrafish guts were captured using the fluorescent microscope, and the IOD was determined (IOD2). The intestinal efflux efficiency (IEE) was calculated according to Equation (1) [47].

IEE = (IOD0/IOD1 − IOD2)/(IOD0/IOD1)(1)

### 4.6. Peristalsis-Promoting Effect

In this experiment, AB zebrafish embryos at 3 dpf were randomly divided into six groups and colitis zebrafish was induced as described in Section 4.4, for a total of 6 groups with 4 repeat holes per group (10 per well). Then, each group was stained with 0.2% calcein for 1.5 h. After that, the zebrafish were rinsed three times in PBS. The peristalsis-promoting effect was evaluated according to intestinal peristalsis frequency of each zebrafish in 1 min, which was recorded by an Olympus SZX16 fluorescence microscope (Tokyo, Japan).

### 4.7. Pathological Observation and Ultrastructure of the Intestinal Tissue of Zebrafish

The pathological observation of the intestinal tissue of zebrafish was examined by using the hematoxylin and eosin (H&E) [48] and Alcian blue (AB) [49] methods. In this experiment, AB zebrafish embryos at 3 dpf were randomly divided into six groups and colitis zebrafish was induced as described in Section 4.4. Following drug treatment, ten zebrafish larvae were randomly collected from each group and fixed in 4% paraformaldehyde for 24 h. Then, the fixed larvae were dehydrated in gradient ethanol, immersed in xylene, and embedded in paraffin. The tissue sections were observed and photographed under the Olympus SZX16 fluorescence microscope (Tokyo, Japan).

The ultrastructure of the intestinal tissue of zebrafish was observed by using the scanning electron microscopy method [49]. After drug treatment, ten zebrafish larvae were randomly collected from each group and fixed in an electron microscope fixation liquid containing 5% glutaraldehyde. Then, the fixed larvae were dehydrated in propanol, embedded in epoxy resin, and double stained with uranium acetate and lead citrate. Finally, ultra-thin intestinal tissue sections were observed by using the HT7800/HT7700 projection electron microscope (HITACHI, Chiyoda, Japan).

### 4.8. Differential Expression and Enrichment Analysis of RNA-Seq

The wild-type AB strain was used in this experiment. Zebrafish were randomly divided into blank group, modeling group and 80 μg/mL drug administration group, with three repeat holes in each group (30 zebrafish per hole). RNA-sequencing analysis was performed on zebrafish larvae samples with the assistance of OE Biotech Co., Ltd. (Shanghai, China). The differential expression analysis of two conditions/groups (two biological replicates per condition) was performed using the DESeq2 R package (1.20.0). Genes with an adjusted *p*-adjust ≤ 0.05 and absolute fold change ≥ 2 found by DESeq2 were assigned as differentially expressed. The Gene Ontology (GO) enrichment analysis of differentially expressed genes (DEGs) and test the statistical enrichment of differential expression genes in the Kyoto Encyclopedia of Genes and Genomes (KEGG) pathways [50,51] were performed on an online OECloud tools (https://cloud.oebiotech.cn, accessed on 13 February 2023).

### 4.9. Qualification of Gene Expression

The transcripts of several genes involved in the key signaling pathways—*mmp9*, *il8*, *il1β*, *il12*, *il10*, *ripk3* and *bax*—were selected and identified by qRT-PCR. Moreover, the expression levels of key genes in inflammatory-related signaling pathways were also determined. Total RNA was extracted from drug-treated zebrafish samples using the SPARKeasy RNA isolation kit (Sparkjade, Qingdao, China) according to the manufacturer’s instructions. cDNAs were generated using the SPAPKscript II RT Plus Kit (Sparkjade, Qingdao, China) with a random primer, and quantitative RT-PCR reactions were carried out using the ChamQ Universal SYBR qPCR Master Mix. In this study, *β-actin* was chosen as a standard control gene. The sequences of primers in RT-qPCR were listed in Table S6. The RT-qPCR amplification reaction conditions were as follows: 95 °C for 1 min followed by 40 cycles of 95 °C for 20 s and 72 °C for 30 s. The relative mRNA expression changes relative to *β-actin* were calculated using the 2^−ΔΔCT^ method. Relevant primers were designed against zebrafish genes.

### 4.10. Molecular Docking

In the docking study, the key target proteins in the NOD-like receptor signal pathway and necroptosis pathway were chosen as the receptors, and the active peptide and positive control were selected as ligands. The three-dimensional (3D) crystal structure of PYCARD (6KI0), IL-8 (6WZL), IL-1β (7Z3W), Caspase-9 (5WVC) and RIPK1 (7YDX) were obtained from the Protein Data Bank (https://www.rcsb.org/structure/1O8A). The 3D structure of LLTRAGL and 5-ASA were constructed and the energy minimized by using the MM2 molecular mechanics method with Chem3D Pro 14.0 (CambridgeSoft Co., Waltham, MA, USA). Before docking, water molecules and other irrelevant ions were removed from the structure of protein receptors. Subsequently, the receptors and two ligands were energetically minimized by the CHARMm force field. The automated molecular docking studies at the receptor binding sites were performed using the CDOCKER module [12]. The binding site sphere of the PYCARD was set as x: −37.5115, y: 7.0317 and z: 40.2425. The binding site sphere of IL-8 was set as x: −11.1982, y: 25.1655 and z: 46.2784. The binding site sphere of the IL-1β was set as x: 13.6831, y: 44.2132 and z: 60.4207. The binding site sphere of the Caspase-9 was set to x: 67,3437, y: −68.4981 and z: −12.8687. The binding site sphere of the RIPK1 was set to x: −16.7095, y: −5.2390 and z: 37.9227; the radius was set to 20 Å during the simulation. The values of -CDOCKER ENERGY and -CDCKER INTERACTION ENERGY were considered as an evaluation. The best conformation of the ligand and receptor showed the highest values of -CDOCKER ENERGY and -CDCKER INTERACTION ENERGY.

### 4.11. Statistical Analysis

All the results were expressed as the mean ± SEM, and a one-way analysis of variance (ANOVA) was used to identify each group’s significant differences. The statistical significance was set as # *p* < 0.05, ## *p* < 0.01 vs. the blank control group; and * *p* < 0.05, ** *p* < 0.01 vs. the TNBS group.

## 5. Conclusions

In summary, this study showed that the peptide LLTRAGL, derived from *Rapana venosa*, had significant ameliorative effects on TNBS-induced colitis in zebrafish. The underlying alleviative effect of LLTRAGL might plausibly be due to its regulating effect in the NOD-like receptor signal pathway and necroptosis pathway associated genes. Hence, the peptide might be a promising therapeutic candidate against colitis, which provides valuable information for future application of LLTRAGL in IBD.

## Figures and Tables

**Table 1:** LLTRAGL and 5-ASA, respectively, use CDOCKER to dock the CDocker energy and CDocker interaction energy of five proteins.

Protein Name (PDB ID)	−CDocker Energy (KJ/mol)		−CDocker Interaction Energy (KJ/mol)	
	LLTRAGL	5-ASA	LLTRAGL	5-ASA
PYCARD (6KI0)	122.0260	14.2595	82.2045	15.3231
IL-8 (6WZL)	89.7209	29.0584	59.6621	29.9727
IL-1β (7Z3W)	106.3360	18.3206	69.2347	19.1699
Caspase-9 (5WVC)	112.6820	14.8370	98.2614	15.6957
RIPK1 (7YDX)	138.7620	30.1659	146.1860	31.0330

## Data Availability

The data presented in the current study are available on request from the corresponding author.

## Supplementary Materials

The following supporting information can be downloaded at: https://www.mdpi.com/article/10.3390/md22030100/s1, Table S1: Up-regulation and down-regulation of TOP10 genes in TNBS vs. Control and LLTGRAGL vs. TNBS; Table S2: Common differential genes between TNBS vs. Control and LLTGRAGL vs. TNBS; Table S3: Differentially expressed genes in GO enrichment analysis circle; Table S4: Gene names included in inflammatory reactions in GO enrichment analysis circle (order from top to bottom); Table S5: Corresponding Paths in the Chord Graph of TNBS vs. Control and LLTGRAGL vs. TNBS KEGG Analysis; Table S6: Sequence of Primers Used in qPCR; Figure S1: The safe doses screening of LLTRAGL at 10, 20, 40, 80, 160, 320 and 640 μg/mL; Figure S2: 3D diagrams of LLTRAGL and 5-ASA docked with 6KI0, 6WZL, 7Z3W, 5WVC and 7YDX molecules; Figure S3: 2D diagrams of LLTRAGL and 5-ASA docked with 6KI0, 6WZL, 7Z3W, 5WVC and 7YDX molecules.
