# Supplementary material for: The Peptide LLTRAGL Derived from Rapana venosa Exerts Protective Effect against Inflammatory Bowel Disease in Zebrafish Model by Regulating Multi-Pathways
Source: Mar Drugs. 2024 Feb 22;22(3):100. doi: 10.3390/md22030100 (PMC10972007; doi:10.3390/md22030100)
Supplement: Supplementary file 1 [file marinedrugs-22-00100-s001.zip › marinedrugs-2825101-supplementary.pdf]

Supplementary Materials

Table S1. Upregulation and downregulation of TOP10 genes in TNBS vs Control and LLTGRAGL vs TNBS

TNBS-vs-Control		LLTRAGL-vs-TNBS	
**Up**	**Down**	**Up**	**Down**
hp	taar11	LOC110439586	si:dkey-108k21.15
LOC110438125	v2ra18	LOC100148249	zgc:163040
LOC103910140	LOC103910196	il19l	si:dkey-261m9.6
zgc:162999	LOC108191611	LOC108191752	xcr1b.2
ifit15	LOC110439303	si:ch211-161n3.3	si:dkey-210i3.1
LOC100537673	slc2a9l1	LOC101885672	hsd17b1
LOC103909700	LOC101883276	LOC110439111	LOC100005207
plac8.2	LOC110438230	LOC798286	si:ch211-113j13.2
saa	si:dkey-23a13.17	LOC110438230	galnt8a.2
LOC799681	LOC799681	zmp:0000000951	LOC100537673

Table S2. Common differential genes between TNBS vs Control and LLTGRAGL vs TNBS

Gene	TNBS-vs-Control			LLTRAGL-vs-TNBS		
	FoldChange	q-value	Regulation	FoldChange	q-value	Regulation
LOC100005207	13.76383	0.505931	Up	0.037264	0.969268	Down
LOC100332535	0.33165	0.390691	Down	2.891477	0.999748	Up
LOC100333286	0.146747	0.025995	Down	9.705609	0.007064	Up
LOC100537673	37.17435	0.174205	Up	0.026852	0.573022	Down
LOC101882112	0.191244	0.004407	Down	3.454498	0.395912	Up
LOC101882465	0.447066	0.428148	Down	3.016773	0.279018	Up
LOC101883438	0.270038	0.210117	Down	5.524299	0.034646	Up
LOC101884955	3.4649	0.169207	Up	0.247373	0.575013	Down
LOC103911298	0.240568	0.034416	Down	7.700833	4.20E-06	Up
LOC103911768	7.726739	0.368211	Up	0.130317	0.95503	Down
LOC110438230	0.046982	0.44136	Down	21.11199	0.999748	Up
LOC110439345	17.09459	0.403948	Up	0.05843	0.999748	Down
LOC570115	4.299965	0.505931	Up	0.077302	0.474426	Down
bokb	2.093768	0.10603	Up	0.494896	0.421222	Down
ccnd3	12.84518	0.18019	Up	0.18164	0.999748	Down
cd28	0.169753	0.223188	Down	4.525346	0.999748	Up
crybb2	7.178406	0.275846	Up	0.137671	0.752366	Down
egr2a	0.482386	0.000754	Down	0.465768	0.002823	Down
galnt8a.2	27.11974	0.329206	Up	0.036802	0.958294	Down
gck	9.252051	0.480012	Up	0.161531	0.999748	Down
grem1b	0.362372	0.256819	Down	2.278969	0.999748	Up
kcnj1a.3	4.275583	0.000584	Up	2.13613	0.04233	Up
kcnj1a.4	3.124943	0.106646	Up	2.392634	0.160125	Up
nr5a1a	0.141945	0.250112	Down	7.247171	0.768565	Up
rgs2	0.425123	0.360421	Down	2.291183	0.009576	Up
setd1bb	0.411574	0.341259	Down	2.443302	0.999748	Up
si:ch1073-153i20.3	7.921183	0.4873	Up	0.131977	0.999748	Down
si:dkey-162h11.3	0.334929	0.254956	Down	3.543544	0.395912	Up
si:dkey-206d17.12	0.476714	0.233237	Down	2.112704	0.519949	Up
si:dkey-210i3.1	10.32547	0.148439	Up	0.026357	0.164778	Down
si:dkey-78l4.10	16.35614	0.493061	Up	0.061156	0.999748	Down
stx11b.2	0.308546	0.136307	Down	2.742537	0.81103	Up
sult5a1	24.10545	0.000653	Up	4.861383	0.95503	Up
tnfb	3.246856	0.360421	Up	0.358116	0.999748	Down
zgc:162171	0.233836	0.353123	Down	4.675054	0.909583	Up

Table S3. Differentially expressed genes in GO enrichment analysis circle

GOid	Term
GO:0006809	nitric oxide biosynthetic process
GO:0015272	ATP-activated inward rectifier potassium channel activity
GO:0004983	neuropeptide Y receptor activity
GO:0005242	inward rectifier potassium channel activity
GO:0010107	potassium ion import
GO:0002548	monocyte chemotaxis
GO:0048020	CCR chemokine receptor binding
GO:0048247	lymphocyte chemotaxis
GO:0071346	cellular response to interferon-gamma
GO:0071347	cellular response to interleukin-1
GO:0071356	cellular response to tumor necrosis factor
GO:0007160	cell-matrix adhesion
GO:0000786	nucleosome
GO:0006954	inflammatory response
GO:0005244	voltage-gated ion channel activity
GO:0006915	apoptotic process
GO:0034765	regulation of ion transmembrane transport
GO:0006955	immune response
GO:0006813	potassium ion transport
GO:0005525	GTP binding

Table S4. Gene names included in inflammatory reactions in GO enrichment analysis circle (order from top to bottom)

	gene		gene		gene
1	ccl20a.3	41	park7	81	tlr2
2	nos2a	42	tnfrsf21	82	pik3ca
3	tlr3	43	ccl34b.1	83	ccl34a.4
4	si:dkey-23c22.5	44	mapkapk2b	84	ccl19b
5	ccl20b	45	uhrf1	85	si:ch211-195h23.3
6	tnfrsf9a	46	si:dkey-260g12.1	86	fas
7	si:rp71-17i16.5	47	chuk	87	ptgs1
8	cxcl11.6	48	syk	88	cxcl8a
9	rel	49	pik3cb	89	zgc:153759
10	ccl38a.3	50	tollip	90	xcl32a.1
11	nradd	51	ccl34b.4	91	tac4
12	cd40	52	agtr2	92	ptgs2b
13	cxcl20	53	hdr	93	il1b
14	tlr8b	54	elf3	94	ccl25b
15	pycard	55	mpx	95	mst1
16	csf1ra	56	si:ch211-261n11.8	96	tnfrsf11a
17	tlr9	57	thbs1b	97	sgk1
18	cxcl11.7	58	si:ch73-361p23.3	98	ripk2
19	tnfrsf9b	59	chid1	99	mmp25b
20	il17c	60	tnfa	100	mapkapk2a
21	nfkb1	61	gbp4	101	si:ch211-66k16.27
22	il17d	62	ccl34b.8	102	ccl35.2
23	ngfra	63	pik3cg	103	cxcr3.3
24	ccl38a.5	64	si:dkey-23c22.9	104	nrros
25	hrh1	65	ccl19a.1	105	pik3cd
26	ccl19a.2	66	ccl38a.4	106	loxl3b
27	si:ch211-112c15.8	67	lyn	107	ptk2aa
28	rela	68	tnfrsfa	108	si:dkey-42l23.4
29	ccl20a.3	69	cxcl8b.1	109	cxcl11.5

30	il17c	70	ccl33.3	110	zgc:158659
31	tnfrsf1b	71	jak2a	111	si:ch211-261n11.7
32	tlr1	72	mmp25a	112	zgc:153631
33	si:ch211-131k2.2	73	jak2b	113	fpr1
34	ngfrb	74	zgc:153935	114	relt
35	relb	75	tac1	115	cxcr3.1
36	ccl44	76	ccl36.1	116	il17a/f3
37	myd88	77	lta4h	117	il1fma
38	loxl3a	78	ccl25a	118	ptgs2a
39	ikbkb	79	si:dkey-23c22.6	119	ccl38.1
40	nfkb2	80	cxcl18b		

Table S5. Corresponding Paths in the Chord Graph of TNBS vs Control and LLTGRAGL vs TNBS KEGG Analysis

TNBS-vs-Control	LLTGRAGL-vs-TNBS
Amino sugar and nucleotide sugar metabolism	Cytokine-cytokine receptor interaction
Starch and sucrose metabolism	Necroptosis
C-type lectin receptor signaling pathway	Steroid hormone biosynthesis
Cytokine-cytokine receptor interaction	Arachidonic acid metabolism
Hedgehog signaling pathway	Amino sugar and nucleotide sugar metabolism
Phagosome	NOD-like receptor signaling pathway
Cell adhesion molecules	Insulin signaling pathway
NOD-like receptor signaling pathway	alpha-Linolenic acid metabolism
FoxO signaling pathway	Arginine biosynthesis
Steroid hormone biosynthesis	Linoleic acid metabolism

Table S6. Sequence of Primers Used in qPCR

Gene	mer orientation	Nucleotide sequence
β-actin	Forward	GCATTGCTGACCGTATGCAG
	Reverse	ACTCCTGCTTGCTGATCCAC
nod2	Forward	GCAAGGAGGGGGTTGATTGT
	Reverse	TCTGCATTCTTGCTGGCTCA
myd88	Forward	ACGGCTAATCCCTGTCGTCT
	Reverse	CAGATGGTCAGAAAGCGCAG
nlrp1	Forward	AAATGTGGACACCGTTGCAG
	Reverse	ACTGGTTGATTTGTGATGTATGATT
nlrp3	Forward	AGCTCACGTTCATCTGCTTCT
	Reverse	GACCCGTCCATTCAGTCTCC
pycard	Forward	CGCGTCACAAAGTCTGCAAT
	Reverse	TTTGATGTGAACACGCCGAC
nfkb	Forward	AGAGGACAATCTTCCGTCGC
	Reverse	GGGTCTCTACGGGATAACGC
tnfa	Forward	GGAGAGTTGCCTTTACCGCT
	Reverse	CCTGGGTCTTATGGAGCGTG
mmp9	Forward	CATCCGCAACTACAAGACATTC
	Reverse	GGTCCAGTATTCATCGTCATCA
il8	Forward	TCCTGGCATTTCTGACCATCAT
	Reverse	ATGCGTCGGCTTTCTGTTTC
Il1β	Forward	GTGGACTTCGCAGCACAAAA
	Reverse	AAGACGGCACTGAATCCACC
Il12	Forward	CGACAATGAGGGCTCCTACC
	Reverse	ATGTCTGTTCTCACCCCACG
cox2	Forward	ATCATTCTTGGAGCGGTCTACT
	Reverse	TACTCTGAGCGATGACATAGGC
tgfβ	Forward	GAACTCGCTTTGTCTCCA
	Reverse	TACAGTCGCAGTATAACCTCA
Il10	Forward	CGGGATATGGTGAAATGCAAGA
	Reverse	AGAGCAAATCAAGCTCCCCC
caspaase-9	Forward	CTGAGGCAAGCCATAATCG
	Reverse	AGAGGACATGGGAATAGCGT
ripk1	Forward	TGATGTACGAGAGCCACACG
	Reverse	TACGAGCCACTTGCTTCCAG
ripk3	Forward	TTGTCCCGAGTGGCTGAAAA

	Reverse	ACGGCTCCTTCCCAGTGATA
caspase-1	Forward	TCAGCAAAGGAAATGGAT
	Reverse	TTAGACGGCGGTAGACAT
tlr4	Forward	ACACATTGAACCAGGAGCTT
	Reverse	TGTCCACGAAATGAAAGCCG
bax	Forward	TACTTTGCCTGTCGCCTTGT
	Reverse	AGCGAGGAAAACTCCGACTG

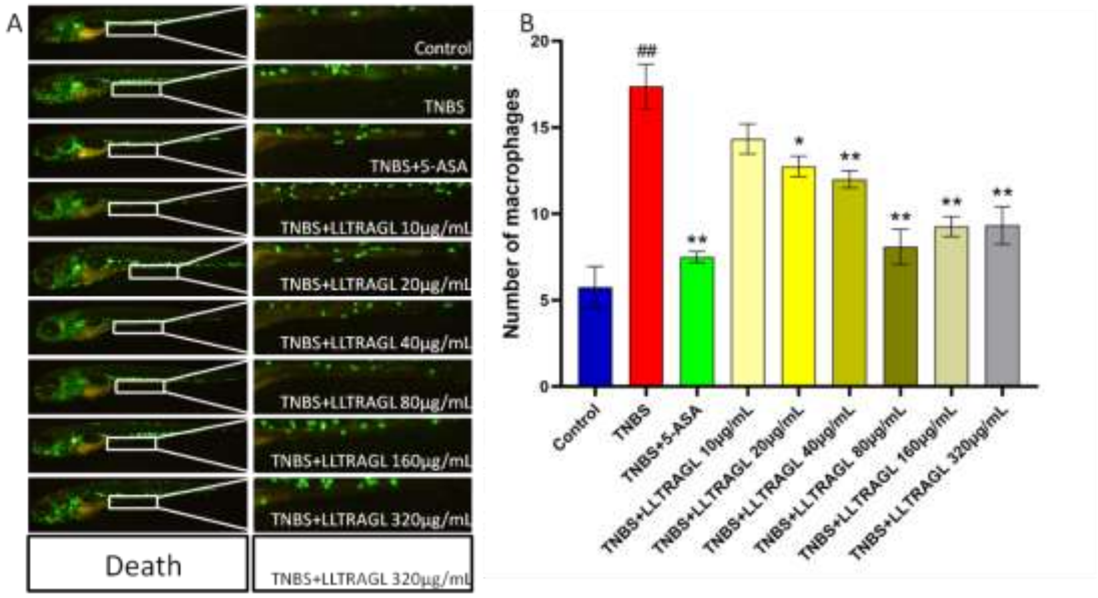

Figure S1: The safe doses screening of LLTRAGL at 10, 20, 40, 80, 160, 320 and 640 μg/mL

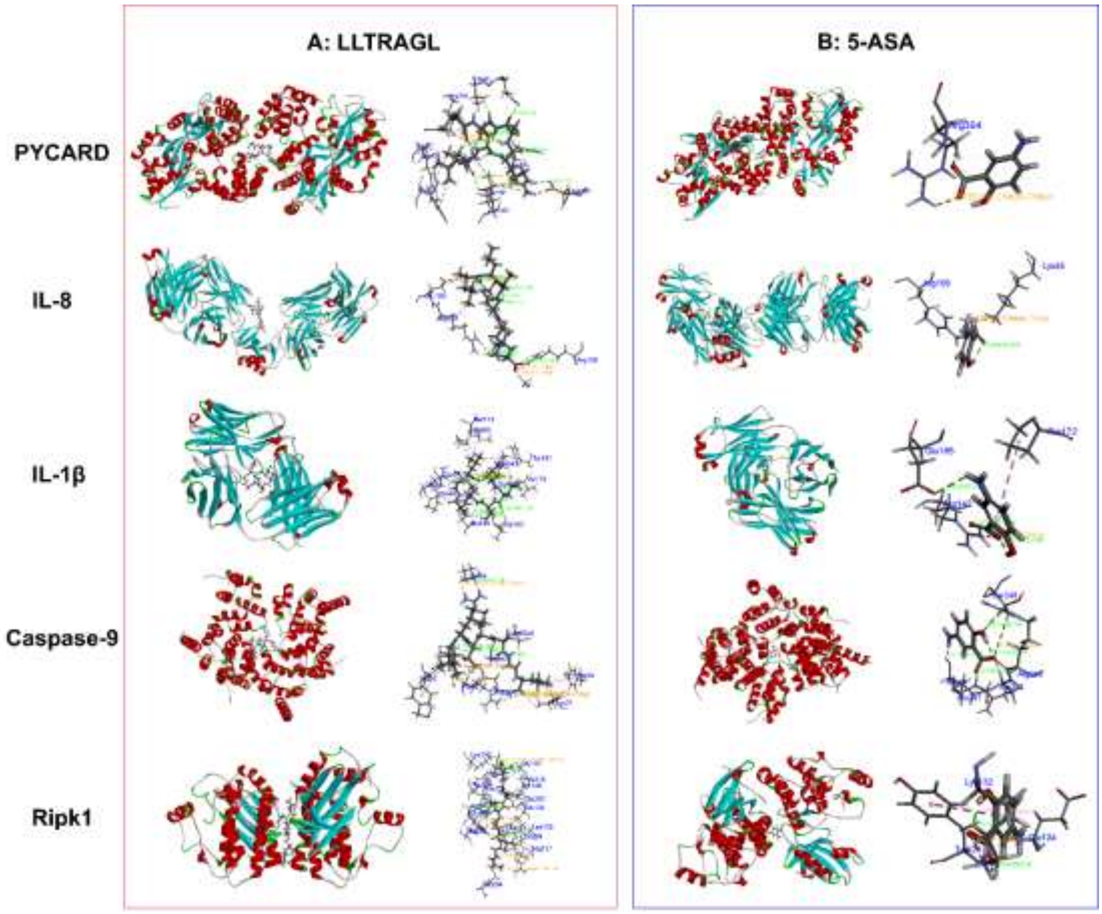

Figure S2. 3D diagrams of LLTRAGL and 5-ASA docked with 6KI0, 6WZL, 7Z3W, 5WVC and 7YDX molecules, respectively

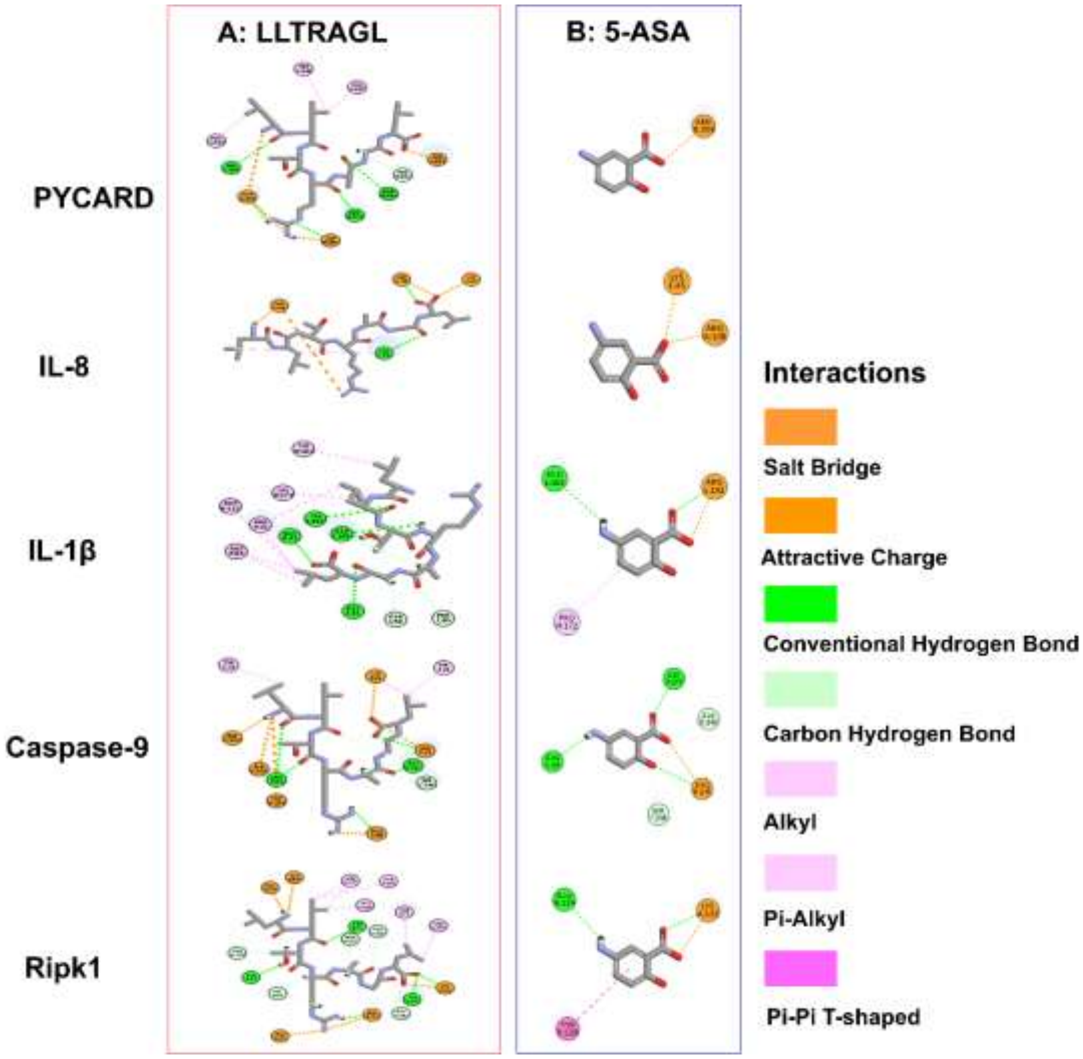

Figure S3. 2D diagrams of LLTRAGL and 5-ASA docked with 6KI0, 6WZL, 7Z3W, 5WVC and 7YDX molecules, respectively
